# Lipopolysaccharide Recognition in the Crossroads of TLR4 and Caspase-4/11 Mediated Inflammatory Pathways

**DOI:** 10.3389/fimmu.2020.585146

**Published:** 2020-11-27

**Authors:** Alla Zamyatina, Holger Heine

**Affiliations:** ^1^ Institute of Organic Chemistry, Department of Chemistry, University of Natural Resources and Life Sciences, Vienna, Austria; ^2^ Research Group Innate Immunity, Research Center Borstel—Leibniz Lung Center, Airway Research Center North (ARCN), German Center for Lung Disease (DZL), Borstel, Germany

**Keywords:** lipid A, inflammation, chemical structure, innate immunity, structural basis, molecular recognition, TLR4/MD-2, aminoarabinose

## Abstract

The innate immune response to lipopolysaccharide is essential for host defense against Gram-negative bacteria. In response to bacterial infection, the TLR4/MD-2 complex that is expressed on the surface of macrophages, monocytes, dendritic, and epithelial cells senses picomolar concentrations of endotoxic LPS and triggers the production of various pro-inflammatory mediators. In addition, LPS from extracellular bacteria which is either endocytosed or transfected into the cytosol of host cells or cytosolic LPS produced by intracellular bacteria is recognized by cytosolic proteases caspase-4/11 and hosts guanylate binding proteins that are involved in the assembly and activation of the NLRP3 inflammasome. All these events result in the initiation of pro-inflammatory signaling cascades directed at bacterial eradication. However, TLR4-mediated signaling and caspase-4/11-induced pyroptosis are largely involved in the pathogenesis of chronic and acute inflammation. Both extra- and intracellular LPS receptors—TLR4/MD-2 complex and caspase-4/11, respectively—are able to directly bind the lipid A motif of LPS. Whereas the structural basis of lipid A recognition by the TLR4 complex is profoundly studied and well understood, the atomic mechanism of LPS/lipid A interaction with caspase-4/11 is largely unknown. Here we describe the LPS-induced TLR4 and caspase-4/11 mediated signaling pathways and their cross-talk and scrutinize specific structural features of the lipid A motif of diverse LPS variants that have been reported to activate caspase-4/11 or to induce caspase-4/11 mediated activation of NLRP3 inflammasome (either upon transfection of LPS *in vitro* or upon infection of cell cultures with intracellular bacteria or by LPS as a component of the outer membrane vesicles). Generally, inflammatory caspases show rather similar structural requirements as the TLR4/MD-2 complex, so that a “basic” hexaacylated bisphosphorylated lipid A architecture is sufficient for activation. However, caspase-4/11 can sense and respond to much broader variety of lipid A variants compared to the very “narrow” specificity of TLR4/MD-2 complex as far as the number and the length of lipid chains attached at the diglucosamine backbone of lipid A is concerned. Besides, modification of the lipid A phosphate groups with positively charged appendages such as phosphoethanolamine or aminoarabinose could be essential for the interaction of lipid A/LPS with inflammatory caspases and related proteins.

## Lipopolysaccharide Detection by the Innate Immune System 

Early detection of pathogen-associated molecular patterns (PAMPs) universally shared by Gram-negative bacteria is a crucial element for the initiation of innate immune responses such as inflammation (1, 2). LPS is a glycan based Gram-negative PAMP that is either expressed on the bacterial cell surface or associated with intracellular or outer membrane vesicles (OMV). LPS prompts the induction of mammalian innate immune responses through a meticulously organized sequential event that starts with the binding of LPS to LPS-binding protein (LBP), transfer to cluster of differentiation-14 (CD14) and, finally, engagement of the germline-encoded pattern-recognition receptor (PRR) Toll-like receptor 4/myeloid differentiation-2 (MD-2) complex (3–6). TLR4 is a type I transmembrane protein entailing a leucine-rich repeats ectodomain, a transmembrane domain and a cytosolic Toll-IL-1 receptor (TIR) domain which is involved in induction of the downstream signaling cascades. MD-2 is a secreted accessory molecule which is physically associated with TLR4 and essential for LPS recognition and binding. LPS-induced homodimerization of ternary TLR4/MD-2/LPS complexes results in the assembly of particular intracellular adaptor protein complexes which leads to the activation of various transcription factors such as NF-κB, followed by induction of expression of cytokines and IFNs. Inadequate regulation of the TLR4 signaling contributes to the pathogenesis of a number of acute and chronic inflammatory as well as autoimmune diseases such as allergy, arthritis (7–9), asthma (10–12), cardiovascular disorders (13), Alzheimer disease-associated pathology (14) and systemic inflammatory response syndrome (SIRS) and septic shock (15, 16). Impressive research demonstrated that down-regulation of the TLR4 mediated signaling can be useful for therapeutic benefits and efficient for management of asthma (17, 18), arthritis, (8) viral infections [influenza (19) and Ebola virus (20)], cancer (21), and sepsis (22). Besides, TLR4-mediated signaling has been demonstrated to promote dendritic cells maturation thereby linking innate and adaptive immunity (23, 24) which features activation of the TLR4/MD-2 complex by TLR4-specific ligands of low toxicity as facile approach for development of novel vaccine adjuvants (25–27).

Whereas detection of extracellular LPS and ensuing immune responses through TLR4 signaling pathway plays a major role in the primary detection of LPS, the recognition of cytosolic LPS by intracellular proteases caspase-4/5 (and their mouse homologue caspase-11) is important at a later stage of severe bacterial infection (28–31). Inflammatory caspases are parts of the non‐canonical inflammasome pathway involved in the initiation of a series of inflammatory effects such as endocytosis, autophagy and oxidative burst (28, 32, 33). Activated caspase-4/5/11 induces NLRP3 inflammasome activation and triggers the secretion of IL-1β and IL-18 *via* caspase-1 mediated processing of pro-IL-1β and pro-IL-18, and pyroptosis accounting for endotoxin-related pathology (34–36). Recent studies have underscored the significance of the non-canonical inflammasome signaling in acute and chronic inflammatory conditions including sepsis (37–39), diabetes (40), atherosclerosis (41), and Alzheimer’s disease (42, 43). Although human caspase-5 has been shown to function similar to caspase-4, it is less studies in respect to LPS recognition. In this review we make emphasis on caspase-4/11 and mention caspase-5 whenever appropriate.

### TLR4-Mediated Signaling Pathways

**Figure 1:**
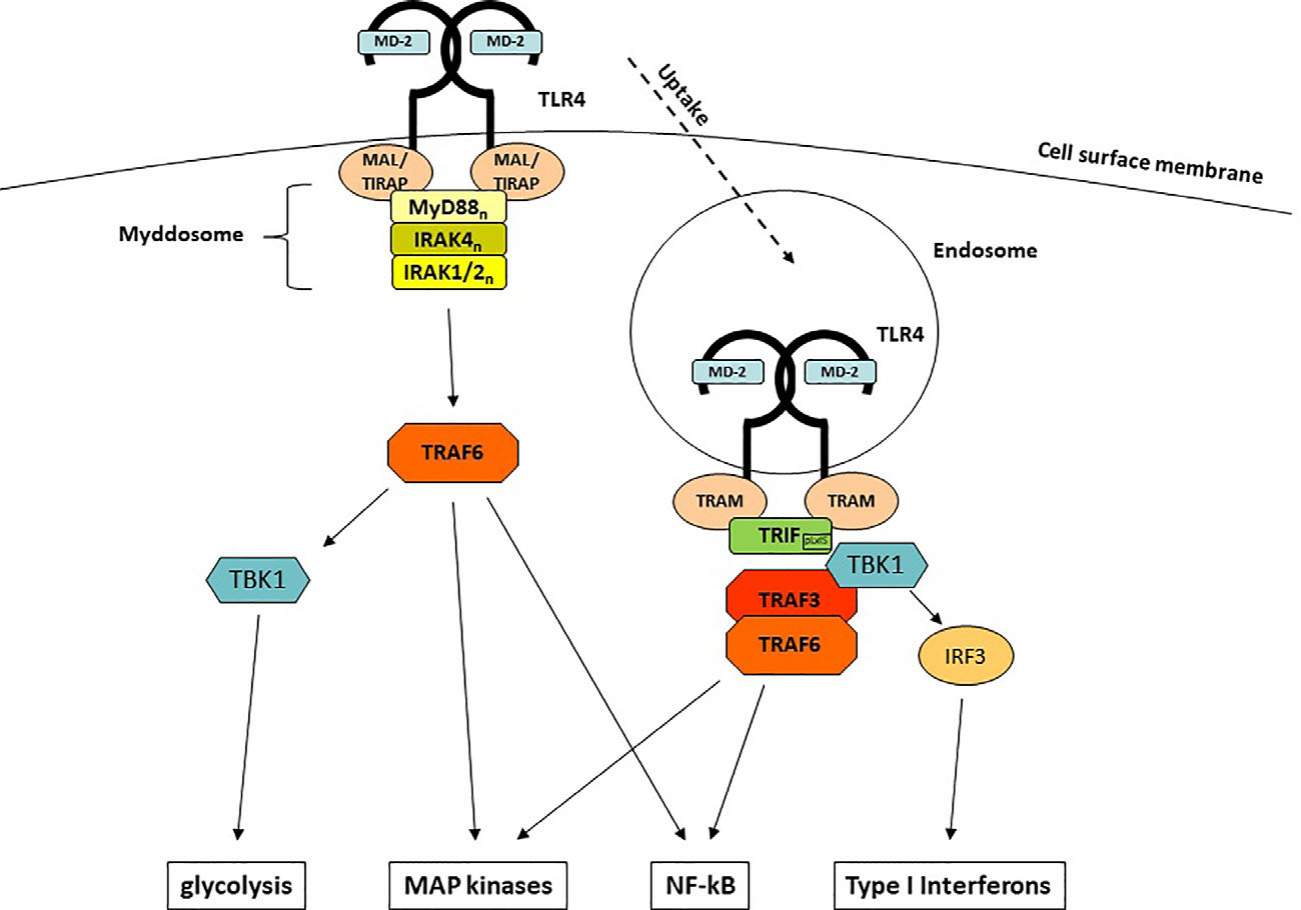
LPS-induced TLR4-mediated signaling pathways.

Upon engagement of MD-2 and TLR4 and the LPS-mediated generation of TLR4/MD-2/LPS homodimers, intracellular signaling is initiated by conformational changes of the Toll/IL-1R (TIR) domain of TLR4. Among all TLRs, TLR4 is unique since it is the only TLR that uses both major signaling adaptors, MyD88 (myeloid differentiation primary response 88) (44) and TIR (Toll IL-1R) -domain containing adaptor inducing Interferon-β [TRIF (45)], as well as the respective adaptor molecules MyD88-adaptor-like [MAL (46)], which is also known as TIR-domain containing adaptor protein [TIRAP (47)] and TRIF-related adaptor molecule [TRAM (48)]. This uniqueness enables TLR4 to induce two different sets of responses: the first set starts at the plasma membrane, depends on MyD88 and leads to a rapid induction of pro-inflammatory cytokines (**Figure 1**). The second set requires internalization, depends on TRIF and emanates signals from endosomal membranes which lead to the induction of a type I interferon response.

MyD88 is a 296 aa adaptor protein containing two major domains: a C-terminal TIR domain which associates with other TIR domain-containing proteins and a N-terminal death domain (DD) which mediates the interaction with the IRAK (IL-1R-associated kinase) family kinases (49–51). Whereas recruitment to TLR4 is facilitated by homotypic TIR domain interactions and requires the bridging adaptor MAL (52, 53), DDs are used to engage members of the IRAK family to the complex. The whole complex comprises of 14-16 MyD88 and IRAK1, -2, and -4 molecules and has been termed the myddosome (54). Such multi-molecular complexes that are functioning as signaling platforms have been termed supramolecular organizing centers (SMOCs) and are crucially important for innate immune signaling (55). The myddosome formation leads to autophosphorylation of the kinase domain (KD) of IRAK4 (56, 57), which in turn activates IRAK1/2. Subsequently, another central (but not exclusive) element of LPS-induced signaling, the E3 ubiquitin ligase TNF receptor-associated factor 6 (TRAF6) (58, 59) associates and gets activated. TRAF6 is central to immune activation (60), as it is able to induce further downstream major signaling pathways that end up in the activation of crucial innate immune transcription factors through TAK1 (NF-κB and AP-1) (61, 62) as well as the induction of glycolysis through TBK1 (63, 64).

Early investigations of MyD88-deficient animals and cells showed that not all LPS responses were completely abolished but rather delayed (44). The reason for the delayed response is the site of its origin: whereas the MyD88-dependent responses are initiated at the cell membrane, the MyD88-independent responses emanate from TLR4-harboring endosomes once MyD88 is discharged from TLR4 (65). The molecule responsible for this surprising effect is TRIF (45, 66). In contrast to TLR3 which can directly recruit TRIF, TLR4 needs assistance from the adaptor protein TRAM (48, 66). Upon binding of TRAM/TRIF to endosomal TLR4, the E3 Ub ligase TRAF3 (67) is recruited and subsequently activates TBK1. Although TBK1 is also part of the MyD88/TRAF6-dependent signaling, it only leads in conjunction with TRIF to the induction of IFNs, the hallmark of the TRIF-dependent immune response: a so-called pLxIS motif in TRIF becomes phosphorylated by TBK1 and can interact with the key interferon-regulatory factor IRF-3 (68), which by itself is another substrate for TBK1 (69, 70). In addition to TRAF3, TRIF can also recruit TRAF6, which explains the delayed NF-κB translocation and MAP kinase activation seen in MyD88-deficient cells.

Most of the LPS-induced cytokines, chemokines and interferons are regulated through the induction of mRNA expression. However, one of the major pro-inflammatory cytokine, IL-1β (as well as its related IL-1-superfamily member IL-18) which regulates a wide array of immune and physiological responses (71), requires an additional step of maturation/processing by caspase-1 (72). The multi-protein complexes facilitating this maturation are another example of SMOCs (s. above) and have been termed inflammasomes (73). The most important inflammasome responsible for TLR4-dependent IL-1β release consists of the processing protease caspase-1, the adaptor protein ASC and the NLR protein NLRP3 (74, 75). The overall production of IL-1β induced by LPS is controlled on several different levels: induction of NLRP3 and IL-1β mRNA (in part through translocation of NF-κB) (76, 77), phosphorylation and ubiquitination of NLRP3 on multiple sites (78–81). Eventually, IL-1β is released by the cells through a process called pyroptosis culminating in Gasdermin D-forming pores in the cell membrane (see also 1.2) (82–84). There are multiple pathways to activate inflammasomes, termed canonical and non-canonical inflammasome activation [reviewed in (85)] and interestingly, LPS shows another species-specific peculiarity: in human cells, LPS is able to induce IL-1β release through an additional inflammasome activating pathway, called alternative activation, which does not require potassium efflux and pyroptosis but uses the TLR4-TRIF axis to activate NLRP3 through caspase 8 (86).

### Caspase-4/11 Mediated Signaling Pathways

Since its discovery in 1999, TLR4 was long believed to be the sole LPS receptor. So it was a surprising finding, when the first reports came out in 2013 showing that intracellular cytosolic LPS—independent of TLR4—was able to trigger noncanonical caspase-11-dependent inflammasome activation that was accompanied by IL-1β release and pyroptosis (28, 34). Subsequently, it was revealed that it is actually caspase-11 and its human orthologs, caspases 4 and 5, that directly bind and get activated by LPS (30, 31, 87).

Binding of LPS by these caspases is mediated by their CARD domain and leads to oligomerization and proximity-induced activation (30, 88). Within this process, auto-proteolysis at Asp285 in the inter-subunit linker of caspase-11 is also required (87). The molecular mechanism by which pyroptosis as well as the release of IL-1β and IL-18 is facilitated was unidentified for many years, despite enormous efforts from multiple groups. Then, in 2015, the long-sought-after molecule was identified as Gasdermin D (82, 89). Gasdermin D belongs to a family of 6 members (based on sequence homology), all harboring an auto-inhibitory carboxy-terminal domain (CTD) linked to the membrane pore-forming amino-terminal domain (NTD). Proteolytically active caspase-11 then cleaves Gasdermin D within the linker region, effectively separating the two domains from each other. Since the NTD has a high affinity to the negatively charged membrane phospholipids such as phosphoinositides and cardiolipin, it localizes to the plasma membrane. Finally, the NTD self-assembles to form pores presumably out of 26-28 NTDs in the plasma membrane which rapidly induce pyroptosis and allow the release of IL-1β and IL-18 (82, 83, 89–94). Caspase‐11 activation induced by intracellular LPS also drives the release of pro‐inflammatory cytokines, interleukin (IL)‐1β and IL‐18 by triggering the activation of the NLRP3 inflammasome. But how is the activation of the NLRP3 inflammasome achieved? Neither caspase-11 nor Gasdermin D do activate the NLRP3 inflammasome directly, but it has been shown that Gasdermin D expression is absolutely required for the activation (89). Due to the size of the NTD pores, they are also non-selective ion channels and thus, enable the efflux of potassium which in itself is known driver of NLRP3 inflammasome activation (95).

### Crosstalk of TLR4- and Caspase 4/11-Dependent Signaling Pathways

The TLR4- as well as the caspase 4/5/11-dependent signaling events induced by LPS are not independent from each other but rather cross-interact at different levels. For example, the expression of caspase-11 is very low under normal conditions, but significantly induced by LPS, whereas the expression of caspase-4 is relatively constant, even in the absence of a priming signal (30). How does LPS induce transcriptional expression of caspase-11? In 2012, the data that TRIF-dependent signaling is licensing caspase-11 for NLRP3 inflammasome activation were convincingly presented (33, 96). This licensing is not mediated by direct interaction of TRIF and caspase-11, but requires Type IFNs. As explained in earlier, TRIF initiates activation of IRF3/7 and the induction of Type I interferon release. The released Type I IFNs then activate in an autocrine/paracrine manner the cell *via* IFNAR1/2-dependent JAK/STAT signaling to initiate pro-caspase-11 expression. In addition, Type I IFNs also drive the expression of GBPs and IRGB10 that are required for caspase-11-dependent responses towards LPS (97–99). Another molecule involved in both the extracellular/endosomal and the cytosolic LPS response is caspase-8. Caspase-8 belongs to the pro-apoptotic caspases and takes part of the alternative inflammasome activation by LPS in human cells (86). However, caspase-8 also cooperates with caspase-11 in the tissues to execute the final steps of endotoxic shock, i.e., tissue injury and cell death (100). The activation of both caspases is cytokine driven, caspase-8 by TNF and caspase-11, as already mentioned, by Type I IFNs, implicating both the LPS/TLR4/MyD88-dependent pathway (TNF) and the LPS/TLR4/TRIF-dependent pathway (TRIF) in this process.

## Recognition of Lipid A/LPS by the TLR4/MD-2 Complex

### Structural Determinants of Lipid A/LPS Guiding Activation of the TLR4/MD-2 Complex in Relation to Virulence

**Figure 2:**
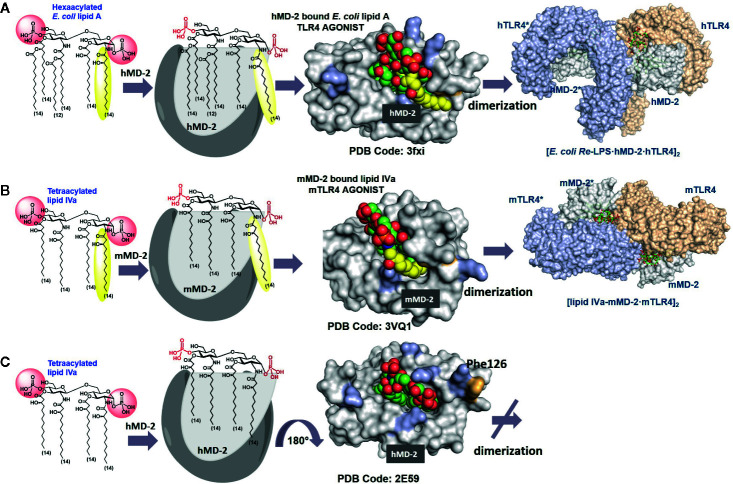
Modes of interaction of agonist and antagonist lipid A variants with the TLR4/MD-2 complex. **(A)** Crystal structure of a binary TLR4·MD-2·LPS complex (PDB code: 3FXI). The 2N-acyl chain (yellow) of the TLR4·MD-2—bound *E. coli* lipid A is exposed on the surface of hMD-2. Phe126 (orange) is directed inward and supports the exterior positioning of the 6th (exposed) lipid chain *via* hydrophobic interactions. **(B)** Species-specific recognition of lipid IVa by mouse TLR4/MD-2 complex. **(C)** Crystal structure of hMD-2 bound lipid IVa, the orientation of the ligand is inverted by 180° compared to agonist ligands (PDB code: 2E59), Phe126 points outward which prevents dimerization with the second TLR4*·MD-2*-ligand complex. Images were generated with PyMol.

LPS is a micro-heterogeneous bacterial glycan which is constituted of three major motifs: the membrane-anchored lipid A, the conserved core oligosaccharide and the variable O-antigen, whereas the lipid A portion exemplifies an “endotoxic principle” of LPS (101–104). Glycolipid “lipid A”–a small (~ 2 kDa) amphiphilic terminal fragment of LPS–is responsible for the activation of the host innate immune response through engagement of two major LPS sensing platforms: transmembrane TLR4/MD-2 complex (105, 106) and cytosolic inflammatory caspases (30). Structurally, lipid A is composed of a polar “head group” and a bulky hydrophobic cluster entailing four to seven long chain 3-hydroxylated lipid residues (107, 108). The polar region of lipid A consists of a β(1→6)-linked diglucosamine backbone which is decorated by two phosphate groups - at position 4´ (P-4′) of a distal GlcN residue and at position 1 (P-1) of a proximal GlcN moiety (**Figure 2**). Positions 2,3 and 2′,3′ of the proximal and the distal glucosamines are usually acylated by the long chain (*R*)-3-hydroxyalkanoic and/or (*R*)-3-acyloxyalkanoic acids. The endotoxic activity of LPS generally relies on the number, length and distribution of lipid chains along the disaccharide backbone of lipid A as well as on the phosphorylation status of the sugar units. A canonical endotoxic lipid A of *E. coli* is hexa-acylated (the lipid chains entail 12 to 14 carbon atoms) and possesses two phosphate groups. The non-endotoxic lipid A variants are usually under-acylated, and/or possess longer (C_16_-C_18_) lipid chains and lack at least one of the phosphate groups. The TLR4/MD-2 receptor complex responds to very low concentrations (picomolar magnitudes) of LPS *via* recognition and binding of distinct structural motifs of lipid A through majorly hydrophobic, but also ionic interactions.

The lipid A binding site on MD-2 is remarkably large and consists of a deep hydrophobic Leu- and Phe- rich cavity, crowned on the top with a number of Arg and Lys residues. Hydrophobic groove of MD-2 can accommodate multiple acyloxy- and acyloxyacyl lipid chains, whereas positively charged side chains at the rim of the binding pocket can establish ionic interactions with the lipid A phosphate groups (5). MD-2 is physically associated with TLR4 and the contact area is designated “primary dimerization interface”. Binding of the lipid A motif of LPS by MD-2 initiates and supports the assembly of a hexameric ligand-receptor complex constituted of two copies of the TLR4/MD-2/LPS homodimer (**Figure 2A**). LPS-induced TLR4 complex dimerization is facilitated by hydrophobic interactions of a specific lipid chain of the MD-2–bound lipid A with the second TLR4* (designated as “secondary dimerization interface”) (6). Upon formation of a [TLR4/MD-2/LPS]_2_ complex, the intracellular TIR domains come into vicinity which triggers the recruitment of a number of adaptor proteins (109). The latter event eventually leads to the assembly of a large macromolecular signaling complex called “Myddosome” that, in turn, triggers diverse pro-inflammatory signaling pathways (110, 111).

Generally, binding of the bisphosphorylated hexaacylated lipid A from *E. coli*–a typical TLR4 agonist - results in the efficient TLR4 complex dimerization and robust activation of the pro-inflammatory signaling, whereas binding of tetraacylated lipid A variants blocks the binding pocket of MD-2 for the interaction with endotoxic LPS, thus rendering these lipid A types to potent TLR4 antagonists (**Figure 2C**) (112, 113). The principal differences in binding modes of agonist and antagonist lipid A include 1) the number of lipid chains (four to five) accommodated in the binding pocket of MD-2; 2) the orientation (binding pose +/- 180°) of the carbohydrate backbone of lipid A within the binding pocket of the co-receptor MD-2; 3) the deepness of insertion of the lipid A molecule into the binding cleft of MD-2 (TLR4 antagonists are accommodated deeper in the binding grove of MD-2 compared to agonist lipid A variants); and 4) ligand-induced rearrangement of MD-2 discriminated by different positioning Phe126 residue (located inward for agonist lipid A and outward for antagonist lipid A).

Accordingly, all four long-chain acyloxy residues of underacylated lipid A variants are entirely intercalated into the hydrophobic binding pocket of human MD-2, the lipid A molecule adopts “inverted” orientation with phosphate group P-4′ facing secondary dimerization interface, and the whole molecule is inserted deeper into the binding pocket compared to agonist lipid A (**Figure 2C**) (112, 113). A number of natural and synthetic antagonist lipid As and analogues were shown to selectively bind to MD-2/TLR4 without triggering receptor complex dimerization (112–114). These compounds are extensively studied as candidates for potential therapeutic inhibition of harmful endotoxic effects induced by TLR4 activation (115–117).

In contrast, TLR4 activating lipid A variants are housed in the binding pocket of MD-2 with the glycosidic phosphate P-1 bordering secondary dimerization interface. Thus, the lipid A molecule is rotated by 180° in the binding pocket of MD-2 compared to antagonist binding mode (**Figures 2A, B**). The binding pocket of MD-2 can accommodate only five lipid chains of hexaacylated *E. coli* lipid A, while the 6^th^ 2*N*-acyl lipid chain (linked to the proximal GlcN residue of the diglucosamine backbone) is excluded from the binding grove and presented on the surface of MD-2 at the site engaged in the dimerization with the second TLR4* (secondary dimerization interface). The expulsion of lipid chain out of the binding pocket and the resulting reorganization of the secondary structure of MD-2 is considered the major driving force of the dimerization process (5, 6). It is now well established that both ionic interactions of the lipid A phosphate groups with the Lys and Arg side chains as well as intermolecular hydrophobic interaction of the exposed 2*N*-acyl chain with the second TLR4* contribute to receptor complex dimerization and formation of the active [TLR4/MD-2/LPS]_2_ hexamer (118–120).

The positioning of the ligand (+/-180°) within the binding cleft of MD-2 appears to be crucial for the expression of a particular biological activity. Thus, tetraacylated lipid IVa acts as antagonist at hTLR4 but performs as weak agonist at mouse (m-) TLR4 wherein it binds in an inverted by 180° orientation (similar to *E. coli* lipid A in the binding pocket of hMD-2) and exposes one lipid chain on the surface of the protein (**Figure 2B**) (6, 121). Species-specificity in ligand recognition by the TLR4 system, which is decisive for transition of *in vivo* data obtained in rodent or other animal models to clinical trials, is still not well understood. In addition to the length and number of lipid chains, the distribution pattern of acyl residues along the glucosamine backbone is decisive for lipid A recognition by the TLR4/MD-2. For instance, lipid A variants having four lipid chains attached at the distal GlcN ring and two lipid chains linked to the proximal GlcN (4 + 2 acylation pattern as in *E. coli*) as well as lipid A variants with the acylation pattern (3 + 3) as in *N. meningitidis* are the most powerful TLR4 activators; penta-acylated lipid As with (3 + 2) acylation pattern are inactive (or weakly active), whereas penta-acyl lipid A having (4+1) acylation pattern retains robust activating potential similar to *E. coli* lipid A (122, 123).

### LPS Is Delivered to the TLR4/MD-2 Complex by the Proteins of the LPS Transfer Cascade

LPS is an amphiphilic molecule that contains a relatively small hydrophobic lipid region retaining LPS in the lipid (bi)layer (in the outer leaflet of the outer bacterial membrane or in the lipid layer of the endosomes/OMVs) and a large hydrophilic carbohydrate portion (inner and outer core, O-antigen) which is decorated by a number of negatively and positively charged appendages such as phosphates, phosphoethanolamines, or amino sugars. Despite its large size and complexity, LPS is recognized by the innate immune system through a fine-tuned molecular mechanism which is extraordinary sensitive to minor variations in the structure of lipid A. Regardless its relative heterogeneity in respect to acylation pattern, lipid A represents the most conserved fragment of LPS. Lipid A has an ability to establish high affinity interactions with a number of proteins involved in the LPS transfer and recognition cascades. Prior to interaction with TLR4, the LPS molecule must be “extracted” from the membrane surfaces and transferred to the binding pocket of MD-2 (124, 125) which requires a successive interaction of LPS with LPB (3), and the GPI-anchored differentiation antigen of monocytes CD14 (4, 126, 127). LBP binds sequentially to LPS micelles and to CD14 to form a dynamic intermediate LBP/LPS/CD14-complex, and accomplishes multiple rounds of LPS transfer to CD14. In turn, CD14/LPS rapidly dissociates from LPB-LPS complex and transfers a single LPS molecule to MD-2/TLR4 *via* a direct physical interaction between LRR13-LRR15 domains of TLR4 with CD14/LPS (4). In addition, CD14 mediates LPS internalization through LPS-induced endocytosis of TLR4/MD-2/LPS complexes which eventually leads to endosome-mediated TRIF-dependent signaling resulting in interferon production as well as in activation of NF-κB (128, 129). Although the crystal structures of LBP and CD14 are available (130–132), and the fine dynamics of the LPS transfer cascade by LBP and CD14 has been recently deciphered (4), the precise atomic mechanism and structural background of the LPS/lipid A recognition by LBP and CD14 are still not fully understood. Whereas LPS binding by LBP involves positively charged patches at the LBP N-terminal domain which could attract the phosphate groups/negative charges of LPS by ionic forces (131), CD14 possesses several hydrophobic cavities surrounded by positively charged side-chains which, most likely, bind LPS through majorly hydrophobic but also ionic interactions (132).

## Interaction of Lipid A/LPS With Inflammatory Caspases

In addition to the activation of a canonical (caspase-1–dependent) inflammasome, LPS mediates the noncanonical (caspase-4/11 – dependent) inflammasome activation when mammalian immune cells are challenged with intracellular bacteria including *Shigella flexneri*, *Salmonella enterica* serovar *Typhimurium* (*S. typhimurium*), *Legionella pneumophila*, *Francisella novicida*, several *Burkholderia* species, and *Chlamydia trachomatis* as well as extracellular bacteria such as enterohemorrhagic *E. coli* (EHEC), *Citrobacter rodentium*, and *Yersinia pseudotuberculosis*. For non-canonical inflammasome activation, the bacterial products such as LPS must be translocated into the host cytosol which can be achieved *via* type III (T3SS) or type IV secretion system (T4SS) abundantly expressed in the infectious strains of several bacteria (31, 133).

### Cytosolic Delivery of LPS for Noncanonical Inflammasome Activation

LPS is a relatively large 20 kDa glycan which cannot cross cellular membranes by itself, so that sophisticated molecular mechanisms are required to deliver or transfect LPS derived from non- cytosolic bacteria into the cytosol of the hosts’ immune cells for non-canonical inflammasome activation. Also, many intracellular bacteria survive within vacuoles and use special protein complexes to let their PAMPs access the host cytosol. It has been proposed that LPS can enter the cytosol through multiple pathways.

Intracellular bacteria which reside and replicate within distinct cellular compartments evolved special secretion systems to allow LPS to access cytosol. For example, *Salmonella* uses type 3 secretion system (T3SS) to invade epithelial cells and to establish vacuolar compartments (SCV, *Salmonella*-containing vacuole), which helps bacteria to survive within phagocytes (133). LPS can gain access to the cytosol through lysis of bacteria-containing vacuoles formed by eukaryotic membranes of the host cells (134, 135). Also *L. pneumophila* usually survives within the vacuole, although certain mutants can atypically enter the cytosol (135). Since many Gram-negative pathogens known to activate caspase-4/11 are not cytosolic, a specific molecular mashinery which allows LPS from these bacteria to gain access to the cytosol for caspase-4/11 activation has been evolved.

One of the plausible mechanisms for LPS internalization and intracellular delivery involves LPS binding by high-mobility group box 1 (HMGB1) - an alarmin which can efficiently transport LPS into the cytoplasm through receptor for advanced glycation end products (RAGE)-mediated endocytosis (17, 136, 137). Through internalization of HMGB1-LPS complexes mediated by RAGE, HMGB1 induces destabilization of lysosomes for cytosolic LPS delivery. HMGB1 was demonstrated to bind LPS *via* LPS-binding domains (the A and B box), although the structural requirements for LPS recognition by HMGB1 are currently unknown (138). TLR4 activation by LPS was shown to induce HMGB1 release from hepatocytes followed by direct LPS binding, and the LPS translocation by induction of lysosomal rupture. Interestingly, HMGB1 has long been supposed to have high affinity to LPS and to interfere with TLR4/MD-2/CD14 signaling (138, 139). Another report describes elevated production of HMGB1 in hepatocytes in response to the LPS-induced TLR4 and caspase-11/Gasdermin D signaling (140) indicating that HMGB1 represents a danger molecule released in response to NLRP3 inflammasome activation.

It has been also suggested that outer membrane vesicles (OMVs)—the naturally secreted products of Gram-negative bacteria—can function as cytosolic LPS delivery vehicles (141). Generally, OMVs promote the induction of pro‐inflammatory mediators *in vivo* during infection with Gram-negative pathogens such as *H. pylori*, *L. pneumophila*, *S. typhimurium* and other (142). The membrane composition of OMVs is rich with LPS required for OMV stability and is very similar to the content of extracellular vesicles formed by eukaryotic cells (143). Recent studies suggest that OMVs can directly transport membrane-associated PAMPs into the host cells where they can be taken up through endocytosis, or act as vehicle for the internalization of LPS into the cytosol (144–147). Furthermore, internalization of LPS-containing OMVs by guanylate-binding proteins (GBPs, interferon-inducible GTPases) promotes localization of LPS in the cytoplasm followed by caspase-4/11 mediated activation of NLRP3 (134, 148). GBPs associate with LPS-containing membrane surfaces and contribute to cytosolic immune detection of LPS by facilitating its interaction with caspase-4/11. GBPs were also shown to assist in disruption of pathogen-containing vacuoles thus allowing LPS of cytosolic bacteria to reach the cytosol (97, 149).

### Guanylate-Binding Proteins as Co‐Factors for Caspase‐4/11 Mediated LPS Sensing

Caspase-4/11 was shown to directly bind to the lipid A motif of LPS, however, lipid A is hidden in the bacterial outer membrane or embedded within the lipid bilayer of liposomal aggregates spontaneously formed by LPS. Therefore, a central question on how the membrane-anchored LPS can interact by its lipid A motif with the CARD of the cytosolic protein caspase-4/11 had to be answered. Recently, guanylate-binding proteins (GBPs) were suggested to govern the recruitment of caspase-4/11 to LPS-rich membrane surfaces.

GBPs play a crucial role in antibacterial defense through modulation of both cell‐autonomous and innate immunity against Gram-negative bacteria (148). The infection of mouse BMDMs with Gram‐negative bacteria induces production of type‐I IFNs which consequently upregulates mGBPs (134, 150). Activation of GBPs also contributes to secretion of IL‐1β and IL‐18, and the induction of pyroptosis through activation of the NLRP3 inflammasome and initiation of molecular mechanisms facilitating LPS release into the cytosol of host cells. GBPs were proposed to aid in the LPS uptake from membrane interfaces und thus, to be involved in the activation of caspase‐11 and the assembly of noncanonical inflammasome (150).

Different roles and functions were suggested to explain GBPs involvement in the induction of proteolytic activity of caspase‐4/11. For instance, in the gut infected with *S. Typhimurium*, GBPs are supposed to contribute to the death and expulsion of infected enterocytes into the lumen. It has been proposed that GBP2‐dependent liberation of *S. typhimurium* LPS into the host cytosol through targeting *S. typhimurium* PCV and promoting its membrane lysis drives caspase‐11‐ and NLRP3‐dependent pyroptosis (134). Apparently, GBP2 contributes to induction of caspase‐4/11 proteolytic activity and noncanonical inflammasome activation in response to infection with *F. novicida* (151), *L. pneumophila* (150) and other cytosolic bacteria (99). GBPs have also been shown to be recruited to cytosolic *S. flexneri* and to prevent spreading of intercellular bacteria by restricting its actin‐driven motility (149). Interestingly, GBPs were degraded over time by *S. flexneri* bacterial proteasomes which were in turn activated by secreted bacterial effectors (152). It has been also reported that GBP recruitment to bacteria such as *Y. pseudotuberculosis* or *L. pneumophila* or their PCVs is dependent on the bacterial type‐3 or ‐4 secretion system, respectively (153, 154).

GBPs were demonstrated to be crucial in mediating caspase‐11 activation in response to outer membrane vesicles (OMVs) from different Gram‐negative bacteria such as *E. coli*, *S*. *typhimurium*, *S. flexneri* or *P. aeruginosa* (97, 98). GPBs were suggested to directly deliver LPS into the host cell cytosol after LPS had been internalized through endocytosis. GBPs could physically associate with cytosolic OMVs upon GBPs isoprenylation, and could govern the activation of caspase‐11 and Gasdermin D *in vivo*. Optionally, OMVs could induce recruitment of GBPs through activation of the TLR4‐TRIF pathway. In all circumstances, LPS was sufficient to initiate GBPs recruitment *in vitro* and GBP deficiency protected against OMV‐induced lethal endotoxemia *in vivo* (98).

Thus, it is by now established that the function of guanylate‐binding proteins is closely linked to their ability to interact with LPS; however, what part of LPS is recognized by GBPs was very long uncertain. Several studies reported on smooth LPS‐induced GBP recruitment to intracellular bacteria which implied the major role of LPS O-antigen in GBPs sensing and the involvement of majorly ionic interactions in this process. Indeed, it was observed that the co-localization of hGBP1 with *S. flexneri* producing LPS-*Ra* mutants was reduced in relation to that of hGBP1 targeting wild‐type bacteria, which insinuated that GBP1 recognizes LPS of *S. flexneri* by its O‐antigen (149). Controversially, hGBP2 was shown to mediate caspase‐4 activation in response to transfection with tetra‐acylated LPS of *F. novicida* (151) which is known to lack the O-antigen and the core sugars (155, 156). This suggests variable sensitivity of different GBPs to particular structural features of LPS and that hGBP2 might contain specific lipid A recognizing motifs. In agreement with the latter observation, it was revealed that caspase-11 activation by transfected lipid A is fully GBP dependent (98) whereas GBPs were only partially required for caspase-11 activation induced by smooth or rough (*Re*-LPS) type *S. minnesota* LPS. Thus, not only the O‐antigen but the lipid A region of LPS could be involved in recognition by GBPs.

Two recent cutting-edge studies independently postulated that the LPS-induced assembly of a GBP coat on the surface of cytosolic *Salmonella* (or on the LPS-rich membrane interface upon cytosolic delivery of LPS) is indispensable for caspase-4 activation (157, 158). Association of GBP1 with the LPS-rich surface of cytosolic *Salmonella* follows bacterial escape from the vacuole and initiates the recruitment of GBP2-4 to assemble a GBP-derived signaling platform. The LPS-induced GBP coating of bacterial surface promotes the recruitment of caspase-4 to the cytosolic face of the GBP coat followed by caspase-4 activation and pyroptosis. Indeed, caspase-4 can efficiently bind to purified LPS and lipid A by its CARD domain *in vitro* but does not bind LPS as a constituent of the bacterial outer membrane in cellular experiments in the absence of GBP. Thus, GBPs could make LPS available for the interaction with caspase-4 by disturbing the integrity of the outer bacterial membrane and making acyl chains of lipid A accessible to their ligand-binding CARD domain.

Further studies are required to understand the structural basis of GBP interaction with lipid A/LPS at the membrane interfaces. The latest findings, however, indicate that GBP1 can directly bind to the LPS coated surface (e.g. outer leaflet of the outer bacterial membrane) and that this interaction is driven by solely ionic forces, whereas the carbohydrate portion of lipid A and the glycan moiety comprising the inner core region are sufficient for LPS-GBP1 interaction (157, 158). All O-antigen and outer core lacking mutants of *E. coli* LPS (*Ra-, Rc-, Rd-, Re*-LPS) could associate with GBP1 and induce GBP1 oligomerization at the LPS-rich membrane interface. Negatively charged groups of the inner core sugars of LPS and the phosphate groups attached at the diglucosamine backbone of lipid A were shown to be crucial in promoting GBP1-LPS interaction (which proceeds presumably through involvement of positively charged surface patch of GBP1) and subsequent activation of the non-canonical inflammasome pathway (158). Remarkably, also LPS from *R. sphaeroides* that acts as caspase-11 antagonist could associate with GBP1 to form higher molecular weight aggregates, which assumes rather broad specificity of GBP1 in recognizing LPS motifs independently on their caspase-4 activity (158). Thus, GBP1 functions as a part of an upstream GBP1-4 complex and orchestrates the recruitment of GBP2-4 to initiate a formation of a signaling platform that is assembled on the LPS-containing membranes. GBP2 and GBP4 are involved in a subsequent recruitment of caspase-4, whereas GBP3 is thought to control its activity (157).

Two alternative modes for GBP-induced caspase-4 recruitment to membrane-embedded LPS have been proposed. A high molecular weight complex formed by GBP-LPS could promote the recruitment of caspase-4 and subsequently transfer LPS onto caspase-4 to trigger its activation. Otherwise, the assembly of GBP-LPS complex on the bacterial surface could disturb the integrity of bacterial outer membrane which would allow an access of caspase-4 to otherwise hidden acyl chains of membrane-anchored LPS (158).

### Structural Features Characteristic to Lipid A of Bacterial Species Inducing Caspase-4/11 Activation: Is There Any Cross-Specificity With TLR4/MD-2?

**Figure 3:**
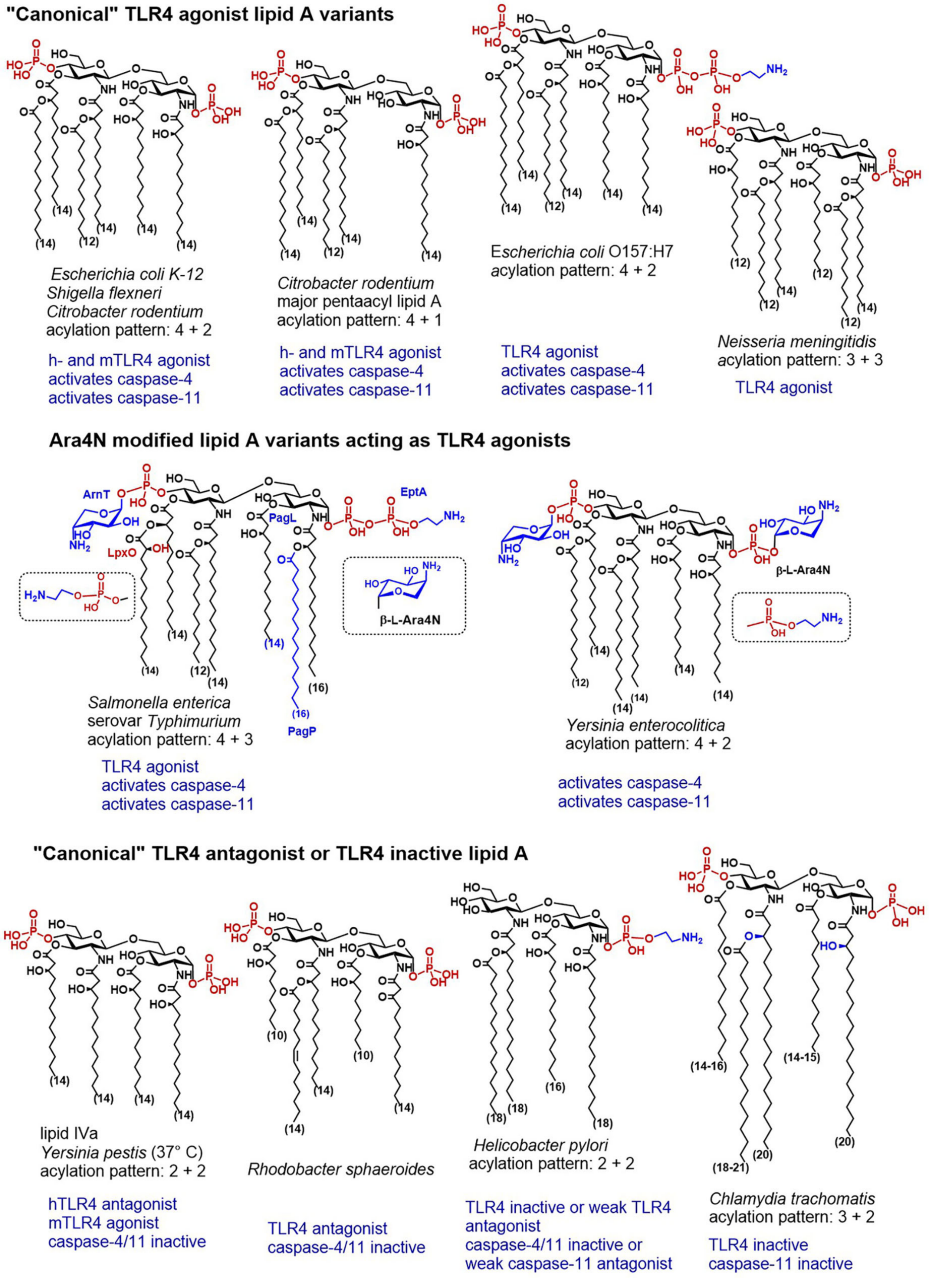
Chemical structures of “canonical” TLR4 agonist and TLR4 antagonist lipid A variants and their caspase-4/11 specific activity (when known).

It has been unambiguously shown that cytoplasmic LPS triggers caspase-4/11-dependent cell death in human 293T cells and mouse macrophages, respectively. Also, LPS–induced caspase-4/11 oligomerization was observed on the pore-limit native gel and the oligomerization was induced by the fully acylated (hexa- to heptaacylated) lipid A fragment of LPS from *S. typhimurium*, *C. rodentium*, *S. flexneri* and *E. coli* (**Figure 3**) (30). Notably, all LPS forms (LPS-*Ra, -Rc, -Rd*, and *-Re*) and *E. coli* lipid A alone could induce caspase-4/11 oligomerization and efficiently stimulated caspase-4/11 activation. Juxtapose, LPS variants bearing fewer lipid chains (LPS from *R. sphaeroides* and biosynthetic precursor of *E. coli* lipid A, lipid IVa) although being able to bind to caspase-11 CARD (caspase activation and recruitment domain) with the affinity similar to hexaacylated LPS, failed to induce caspase-4/11 oligomerization and activation *in vitro*. This was consistent with the reports on the inability of lipid IVa to activate the non-canonical inflammasome in mice (28, 34). Several positively charged residues at the lipid A binding site of CARD were identified indispensable for efficient lipid A—induced oligomerization (30). Interestingly, several lipid A–binding residues (K19, K52/R53, K62/K63/K64) are conserved in caspase-4 but not in caspase-11 which resembles species-specific differences between human and mouse MD-2. Whereas the rim of the binding pocket of human MD-2 is decorated by multiple Lys and Arg residues that are crucial for establishing ionic contacts with the lipid A phosphate groups, mouse MD-2 lacks most of these amino acids (6).

Along these lines, wt strain of *E. coli* induced expression of IL-1β and pyroptosis in wt and TLR4^-/-^ BMDMs, whereas a mutant lipid IVa-producing strain induced TLR4-dependent production of IL-6 (which is consistent with weak agonist activity of lipid IVa at mTLR4), but not the release of IL-1β or pyroptosis in BMDMs (28). Activation of caspase-11 in BMDMs by transfected *E. coli* LPS was supressed when BMDMs were preloaded with underacylated *Helicobacter* lipid A (28) which is also known for its moderate antagonist activity at hTLR4 (159).

Another example of cytosolic bacteria producing LPS with canonical lipid A structure able to potently induce the TLR4-dependent immune responses is *S. flexneri*–a Gram negative pathogen responsible for invasion, disruption and inflammatory destruction of the intestinal mucosa. *S. flexneri* synthesises heterogeneous hexa- to tetraacylated LPS having a “canonical” endotoxin acylation pattern (4 + 2) (**Figure 3**) (160–162). Expectedly, hTLR4 was shown responsive to hexaacylated lipid A variants of *S. flexneri*, whereas mTLR4 was required to elicit TLR4-mediated NF-κB response to penta-acylated mutants (163). Caspase-11 was responsive to LPS introduced by *Shigella* into the cytosol after bacteria escaped the vacuole. *S. flexneri* LPS induced production of IL-1β and pyroptotic cell death resulting in lethal endotoxemia in mice (89). Similarly, infection with *S. flexneri* was responsible for caspase-4 driven inflammatory cell death in HaCaT keratinocytes and human colon adenocarcinoma HT29 cell line (164).

Juxtaposed, LPS from the obligatory intracellular pathogen *C. trachomatis* characterized by a “TLR4 inactive” acylation pattern (3 + 2) of its lipid A (165) failed to activate the non-canonical inflammasome (166). Three out of five acyl chains in *Chlamydia* lipid A exceed the length that is considered optimal (C_12_-C_14_) for the TLR4 activation (**Figure 3**). Both 2- and 2´-*N*-acyl chains as well as the secondary acyl chain at position 2´ of *C. trachomatis* lipid A have a length of up to 21 carbon atoms and the acyl chains that are ester-linked at positions 3- and 3´- are not hydroxylated. All these structural features confer low affinity to human TLR4/MD-2 complex (167, 168) and to the proteins of the LPS transfer cascade (169). Likewise, *Chlamydia* LPS failed to induce the dimerization of mouse TLR4/MD-2 complexes and to activate both NF-κB and caspase-11-mediated signaling in BMDMs (166).

Thus, structure-activity relationships for caspase-4/11 and TLR4 activation seem to be somewhat similar: LPS possessing a “canonical” lipid A structure-hexaacylated/bisphosphorylated-binds to CARD, promotes caspase-4 and caspase-11 oligomerization and induces caspase activation, whereas penta- and tetra-acylated lipid A variants fail to activate caspase-4/11 although can bind to CARD. Similar dependencies were recently demonstrated for synthetic lipid A mimetics close in structure to native lipid A molecules: tetraacylated disaccharide lipid A mimetics (DLAMs) acting as potent TLR4 antagonists did not induce caspase-4/11 proteolytic activity, while synthetic TLR4 agonists (DLAMs having picomolar affinity for TLR4/MD-2) were simultaneously very efficient in inducing oligomerization and proteolytic activity of caspase-4 *in vitro* (170). Intriguing results were obtained for the interaction of DLAMs with caspase-11: despite causing caspase-11 oligomerization, several synthetic lipid A mimicking molecules did not induce caspase-11 catalytic activity which correlated with their chemical structure (170). Thus, the CARD of both capase-4 and caspase-11 was extraordinary sensitive to variations in the primary chemical structure (acylation and phosphorylation pattern) of lipid A mimicking molecules.

All these findings match with a 1:1 ligand-receptor stoichiometry already postulated for the assembly of TLR4/MD-2/LPS complex, which insinuates a high affinity interaction of a single lipid A (or lipid A mimetic) molecule with the CARD. As far as the recognition process is concerned, both the primary chemical structure and the shape of aggregates formed by LPS/lipid A or lipid A mimetic could be involved. Indeed, the latter studies were performed *in vitro* using pore-limit native gel and relatively high lipid A/DLAMs concentrations. Since lipid A/LPS tend to form high molecular mass aggregates in a concentration-dependent manner (171, 172), DLAMs could also form aggregated structures which were, in turn, sensed by the CARDs of caspase-4/11. Such interpretation would be in line with a recent study showing that caspase-4 recognizes LPS-rich membrane interfaces. According to this study, caspase-4 could bind directly to LPS-rich OMVs formed by *N. meningitidis* as well as to the high molecular mass aggregates of purified metabolically radiolabeled LPS (173–175). Indeed, it has been demonstrated that purified caspase-4 (C258A) and CARD domain from *E. coli* could bind huge LPS micelles and disaggregate them to small complexes *in vitro* (176).

Taking into account substantial differences in the molecular and physical properties of monomeric and aggregated structures of lipid A (2 kDa amphiphilic glycolipid) and LPS (20 kDa heterogeneous glycan) and the fact, that lipid A alone could induce pyroptosis *in vivo* (30) and could bind to CARD *in vitro*, we assume that the recognition of particular chemical entities of lipid A is essential for caspase-4/11—LPS interaction. Considering that lipid A is buried within the lipid bilayer to anchor LPS in the membranes or other liposomal interfaces and, therefore, not freely available for the interaction with proteins, an intermediate step preceding lipid A/LPS-CARD interaction with involvement of additional proteins that can extract LPS from the membrane surfaces and deliver the lipid A fragment to CARD can be supposed. For example, TLR4/MD-2 complex “exploits” accessory proteins LBP and CD14 to let the lipid A portion of LPS being directly “delivered” to the binding pocket of MD-2. Recent studies disclosed a fine-tuned mechanism of LPS sensing by inflammatory caspases with involvement of GBPs as supplementary proteins having high affinity for LPS (98, 136, 157, 158). Similar to species-specific recognition of lipid A by the TLR4 system, some not yet fully understood species-dependent differences in the activation of human caspase-4 and mouse caspase-11 by LPS have been observed.

## What Are the Structural Determinants Crucial for LPS/Lipid A Recognition by Inflammatory Caspases?

To explore structure-activity relationships and to establish primary molecular signatures recognised by caspase-4/11 and involved in the non-canonical inflammasome activation, we analyzed the relevant literature from the “chemical” perspective with a special emphasis on particular structural features of LPS/lipid A mentioned in the studies on caspase-4/11- and/or GBPs-mediated inflammasome activation. Remarkably, except for “canonical” hexaacylated lipid A of *E. coli*, *S. flexneri* and *C. rodentium*, the major lipid A species able to induce caspase-4/11 activation are characterized by specific lipid A modifications such as substitution of the phosphate groups by positively charged appendages (phosphoethanolamine or amino sugars) and by a specific acylation pattern (penta- to heptaacylated with fatty acids length up to 18 carbon atoms).

Covalent attachment of positively charged appendages to the phosphate groups of lipid A is considered a part of survival strategy of opportunistic Gram‐negative bacteria. One of the most abundant phosphate group modifications-attachment of ethanolamine (*Helicobacter*) or phosphoethanolamine PNEt (*EHEC, Salmonella*)—is associated with bacterial resistance to cationic antimicrobial peptides (CAMPs) (177). In some species, the phosphate groups of lipid A are substituted by cationic amino sugars-4‐amino‐4‐deoxy‐β‐l‐arabinose (β‐l‐Ara4N) in *Burkholderia, Pseudomonas, Yersinia* or *Salmonella*, or by galactosamine (*Fransicella*) or glucosamine (*Bordetella*) (178–181). Inducible addition of β‐l‐Ara4N to the phosphate residues of lipid A is an adaptive mechanism that assists Gram-negative bacteria to oppose neutralization by CAMPs and to circumvent induction of the innate immune responses in the infected host. Despite rigorous research efforts, no explicit correlation between the presence of β‐l‐Ara4N as a lipid A phosphate group modification and the modulation of TLR4-dependent inflammation could be established (182, 183). To better comprehend the interrelation of caspase-4/11 activation and specific acylation and phosphorylation pattern of lipid A, we provide a short exposè on structural features of lipid A produced by bacterial species that are known to induce caspase-4/11-mediated inflammasome activation and/or TLR4 dependent signaling with special emphasis on LPS remodeling.

### Activation of Inflammatory Caspases by Extracellular Bacteria That Produce TLR4-Agonist LPS Variants

Caspase-4 activating *E. coli* strain O157:H7 (enterohaemorrhagic *E. coli*, EHEC) produces 1-O-P-PNEt lipid A which differs from a “classic” lipid A (hexaacylated, bis-phosphorylated, to one-third substituted with pyrophosphate at position 1)synthesised by *E. coli* serotypes K12 and O111:B4. The occurrence of the phosphoethanolamine modification at the glycosidic phosphate group P-1 in EHEC has been distinctively confirmed (184). EHEC and EPEC (enteropathogenic *E. coli*) strains were shown to activate caspase-4 (133, 164), to induce caspase-4/11-mediated IL-1β and IL-18 secretion and inflammatory cell death, whereas specific T3SS effector protein could inhibit caspase-4/11-dependent inflammasome (185, 186). Whether 1-O-P-PEtN motif of the lipid A fragment of LPS is functionally involved in EHEC-induced caspase-4/11 dependent inflammasome activation remains for now unknown.

*C. rodentium* is a murine Gram-negative bacterium used as a surrogate to study human non-invasive gastrointestinal pathogens EPEC and EHEC since it causes similar transmissible diarrheal disease in mice. The lipid A acylation pattern of *Citrobacter* LPS is identical to that of *E. coli* with exception of relatively high proportion of the penta-acylated species having both 3+2 and 4+1 acylation pattern, whereas the latter is more abundant (**Figure 3**) (187). In agreement with the known principle for “TLR4 agonist” acylation pattern of lipid A, *C. rodentium* induces rapid TLR4-dependent responses in intestinal epithelium, although TLR4- mediated pro-inflammatory signaling is not host-protective and contributes to pathology and morbidity during infection (188). TLR4 was demonstrated particularly important for NLRP3 inflammasome activation in *C. rodentium* and *E. coli* infected mouse macrophages. Importantly, the TLR4/TRIF axis—regulated expression of caspase-11 was indispensable for *E. coli*- and *C. rodentium*—induced NLRP3 inflammasome activation in macrophages (96). Thus, extracellular enteric bacteria must be recognized by both TLR4- and caspase-11 to induce the non-canonical inflammasome activation and pyroptosis. Like pathogenic human-specific *E. coli* strains EPEC and EHEC, *C. rodentium* modifies the phosphate groups of its lipid A with phosphoethanolamine. This covalent modification is primarily catalysed by specific transferases PmrC and CptA, the expression of which is regulated by PmrAB. Interestingly, PEtN modification contributed to maintenance of OMV integrity, but simultaneously negatively affected the rate of production of OMV by *C. rodentium* (189).

*Yersinia* species evolved many strategies to evade the recognition by the human innate immune system, including inducible LPS remodeling. To achieve a suppression of local and systemic inflammation, *Y. pestis* modifies the acylation degree of the diglucosamine backbone of its lipid A from hexaacylated (hTLR4 agonist) to tetraacylated (inactive or hTLR4 antagonist). Thus, the lipid A produced by *Yersiniae* in mammalian host at 37°C is underacylated and similar in structure to lipid IVa which deprives *Yersinia* LPS the hTLR4-mediated activity (**Figure 3**) (190). Mutants producing hexaacylated lipid A (normally synthesised by bacteria in a vector host at 25°C) have been shown to strongly activate the innate immune response in a TLR4-dependent manner (191). Recognition of lipid A by caspase-11 might follow similar structure-activity relationships: transfection of hexaacylated LPS from *Y. pestis* grown at 25°C induced **c**aspase-11-mediated cytotoxicity in mouse macrophages whereas transfection of tetra-acylated LPS from bacteria grown at 37°C did not (34).

**Figure 4:**
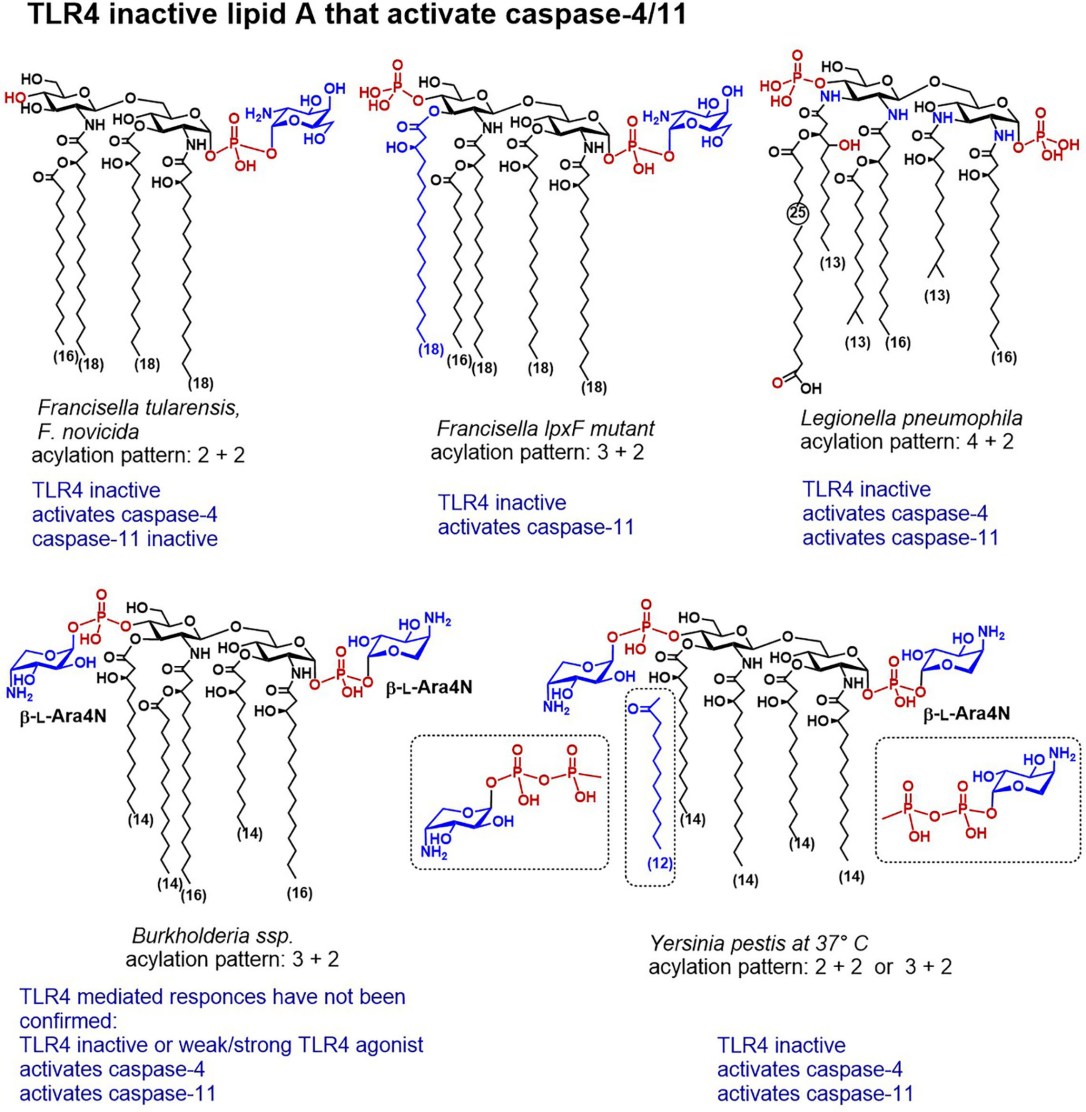
Chemical structures of lipid A variants that do not activate TLR4/MD-2 but are distinguished by a confirmed caspase-4 or caspase-11 mediated activity.

Apart from *Y. pestis*—a facultative intracellular Gram-negative bacterium, and causative agent of bubonic plague, two other *Yersinia* species: *Y. enterocolitis* and *Y. pseudotuberculosis* are pathogenic to humans and cause foodborne infections leading to gastroenteritis and septicemia. The structure of *Y. pseudotuberculosis* lipid A is the closest to *Y. pestis*, as far as the acylation pattern and Ara4N modification is concerned (192). The mechanism of Ara4N modification of lipid A *Y. pestis* is different and more complex than in other bacteria, and the Ara4N modification has been shown to play a crucial role in both transmission and survival of *Y. pestis* in its flea vector and in pathogenicity to human host (193, 194). Both or one phosphate groups of *Y. pseudotuberculosis* lipid A are covalently substituted by Ara4N, and the C_14_ acyloxyacyl chain at position 2 is esterified by palmitoylation (C_16_) (**Figures 3**, **4**). *Y. enterocolitica* entails a shorter secondary acyl chain in position 2 (C_12_ or C_14_). The functional role of inducible addition of Ara4N to the phosphate residues of lipid A in *Yersinia* as well the propensity of its LPS to induce caspase-4/11-mediated pyroptosis has not yet been adequately studied. It has been recently reported that *Yersinia* infection induces caspase-8 mediated pyroptosis which proceeds through cleavage of Gasdermin D, although the involvement of LPS in this process has not been illustrated (195, 196). Temperature-regulated remodeling of lipid A in *Yersinia* substantially complicates studies of *Yersinia* LPS-induced pathogenicity *in vivo*, since tetraacylated lipid A variants produced by *Yersinia* at 37°C might act as TLR4 antagonists in human, simultaneously performing as weak TLR4 agonists in the mouse system. Indeed, the induction of the TLR4/MD-2/LPS—mediated protective responses in mice was responsible for reduced sensitivity of rodents to *Yersinia* infection and indicated a necessity for exploring *Yersinia* virulence factors in humanized mouse models (197).

### LPS-Induced TLR4- and Caspase-4/11-Mediated Responses to Intracellular Gram-Negative Bacteria That Escape the Vacuole

*Salmonella* and *Legionella* are intracellular enteric pathogens known to cause gastroenteritis that can result in a systemic disease. These strains were shown to release specific bacterial antigens into the host cell cytosol and to trigger inflammasome activation within epithelial cells and macrophages (134, 198).

The disaccharide backbone of *L. pneumophila* lipid A contains two 2-diamino-2-dideoxyglucose (GlcN3N) residues instead of common 2-amino-2-deoxyglucose (GlcN) and the four amino groups are substituted by two long-chain (C_16_) β-hydroxyacyl residues, one of which is further acylated by a branched C_14_ lipid chain, whereas the amino group in position 3´entails unusual long-chain (C_25_) fatty acid (**Figure 4**) (199, 200). According to the well-establised structure-activity relationships, lipid A having acyl chains longer than C_14_ have usually much lower affinity to MD-2/TLR4 complex and to LBP than lipid As with a “classic” C_12_-C_14_ acylation pattern. Indeed, the unusual structure of *L. pneumophila* lipid A accounted for the absence of TLR4-dependent endotoxic activity, due to a failure to interact with both TLR4/MD-2 complex and CD14 (201). Although *L. pneumophila* harbors a gene conferring resistance to cationic antimicrobial peptides, the modification of the phosphate groups of its lipid A with cationic residues has not yet been confirmed by structural analysis (202). Infection with *L. pneumophila* induced rapid caspase-11-mediated pyroptosis in mouse macrophages which has been accelerated by deletion of a specific effector supporting the integrity of *Legionella*-containing vacuole (135, 203).

*Salmonella* can alter the structure of its lipid A and remodel the content of the bacterial outer membrane by using several regulatory systems that govern phosphate group modifications crucial for resistance to CAMPs (204). Complex mechanisms which involve several regulatory proteins are implicated in the control of these processes. *S. enterica* serovar *Typhimurium* strains possess specific two-component systems that can add β-l-Ara4N to the lipid A phosphate groups or induce 3-*O*-deacylation at the proximal GlcN residue (**Figure 4**). The former modification promotes bacterial resistance to antimicrobial peptides, whereas the latter enhances the host recognition of lipid A by TLR4 (205). In particular, the PhoPQ two-component system regulates PagP-catalyses addition of a secondary palmitate residue at position 2 of the proximal GlcN moiety, and PagL-induces 3-*O*-deacylation, whereas PmrAB regulates the addition of Ara4N and phosphoethanolamine (206–208). Addition of Ara4N to lipid A inhibits the enzymatic activity of PagL which results in the synthesis of heptaacylated lipid A with Ara4N-modified phosphate groups (207). Heptaacylated *S. Typhimurium* lipid A has reduced hTLR4 activating potential, whereas its 3-*O*-deacylated counterpart (acylation pattern 4 + 2) belongs to the most powerful activators of hTLR4. Juxtaposed, in mouse TLR4 system heptaacylated *S. Typhimurium* LPS isolates induce robust IL-6 production in BMDMs (28).

Not surprisingly, *Salmonella* is able to activate the non-canonical capase-4 and caspase-11 dependent inflammasomes *via* intracellular LPS sensing (28, 133, 209). Whether and how the structure of *S. Typhimurium* lipid A and the presence of positively charged appendages at the phosphate groups influence caspase-4/11—LPS interaction remains to be determined.

#### Structural Peculiarities of *Francisella* LPS Accountable for Species-Specific Caspase-4/11 Activation and TLR4 Escape

The major lipid A of *Francisella* possesses a unique tetraacylated structure lacking the 4′-phosphate group and the 3′-acyl chain and containing an α-d-GalN residue at the glycosidically linked phosphate group (**Figure 4**) (210). All subspecies of genus *F. tularensis* (*Schu S4, holartica*, live vaccine strain LVS (attenuated type B strain), as well as a nonvirulent laboratory strain *F. novicida*) retain analogous lipid A modified with phosphodiester linked GalN at the glycosidic phosphate group P-1 (180, 211–213). The modification of *Francisella* lipid A phosphate residue with GalN is associated with augmented bacterial virulence and resistance to CAMPs, although the consequence of this modification for *Francisella* LPS/lipid A recognition by the innate immune system of the host has not yet been fully clarified. Mutants deficient in GalN modification were shown to induce activation of the innate immune responses in mice and to have weakened pathogenicity (214).

*Francisella* LPS escapes the recognition by both TLR4/MD-2 complex and LBP due to its hypoacylated structure which is assembled in a temperature-dependent manner (215), the inappropriate length of its four lipid chains (C_18_-C_16_) and the absence of a phosphate group at the distal GlcN moiety (P-4´) (216–218). Also, penta-acylated *Francisella lpxF* mutant failed to activate TLR4 which was explained by a non-optimal length of its acyloxy- and acyloxyacyl chains, although this mutant displayed attenuated virulence. Thus, the structural features of *Fransisella* lipid A do not comply with the well-establish requirements for the lipid A sensing by TLR4/MD-2. The lack of TLR4 stimulating potential of tetraacylated *Francisella* LPS is compensated by the activation of NOD-like receptors (NLRs), and the adaptor molecule ASC which are involved in the regulation of caspase-1-mediated inflammasome activation (219, 220), as well as by the GBPs-promoted activation of AIM2 (Interferon-inducible protein AIM2 also known as “absent in melanoma 2”) inflammasome in mice (221, 222). Thus, TLR4 signaling plays comparatively insignificant role in defence against *Francisella* infection or in protection after administration of live vaccine strain LVS in mice (223, 224).

The nonvirulent laboratory strain *F. novicida* as well as several other *Francisella* strains exhibit a truncated lipopolysaccharide form deprived of the polymeric O-antigen and the core sugars. A bifunctional Kdo-hydrolase, an LPS remodeling enzyme responsible for the synthesis of truncated LPS structures, has been identified in the inner membrane of *F. novicida* (155, 156, 225, 226). Thus, around 90% of *F. novicida* LPS consists of a solely tetraacylated lipid A modified with α-d-GalN at the glycosidic phosphate group P-1 (**Figure 4**) (227). A recent study postulated that tetra-acylated LPS/lipid A of *F. novicida* can be detected by caspase-4 upon LPS transfection in human monocyte-derived macrophages (151). Although transfected *F. novicida* LPS was 10-fold less potent compared to (transfected) *E. coli* LPS to induce activation of caspase-4, the innate immune responses to *F. novicida* LPS (i.e. IL-1β release and cell death) were fully caspase-4 driven. Importantly, these responses were essentially GBP2-dependent, highlighting a crucial role of guanylate binding proteins in facilitating recognition of cytosolic lipid A/LPS structures by inflammatory caspases. Similar to the TLR4/MD-2 complex, caspase-4 and caspase-11 exhibit species-specific differences in sensing underacylated lipid A which escapes caspase-11 recognition. The disparities in sensing structurally different lipid A molecules might be due to dissimilarities between the CARD domains of caspase-4 and caspase-11, which share 51% identity. Accordingly, tetraacylated *Fransicella* lipid A could not be detected by caspase-11 after *F. novicida* LPS transfection in mouse macrophages (34). However, transfection of penta-acylated *Francisella* LPS (*lpxF* mutant) that retains the phosphate moiety at position 4´ and the *N*-linked C_16_-C_18_ fatty acid at position 3´ of the diglucosamine backbone (210, 228) resulted in a robust caspase-11 activation followed by pyroptosis. Thus, caspase-4 seem to be more receptive to the number of phosphate groups decorating the diglucosamine backbone of lipid A than to the acylation pattern, both in respect to the length and number of acyl chains. These data provide unequivocal evidence for the primary role of lipid A in driving the activation of caspase-4/11 and for apparently high affinity interaction of particular structural elements of lipid A with caspase-4/11 CARD.

### Detection of Ara4N Modified *Burkholderia* LPS in the Cytosol of Mammalian Cells

The lipid A phosphate groups of clinical isolates of *Burkholderia* are substituted by an amino sugar β‐l‐Ara4N that is believed to reduce the net negative charge of the bacterial membrane and confer resistance to antibiotics. Host-adapted *Burkholderia* species cause severe pneumonia and systemic endotoxemia in cystic fibrosis and melioidosis patients which is linked to a potent cytokine-inducing capacity of *Burkholderia* LPS. Substitution of both phosphate groups with Ara4N was confirmed for ubiquitous environmental *Burkholderia* strain *B. pseudomallei*—an opportunistic facultative intracellular pathogen causing melioidosis in humans, as well as for a less virulent strain *B. thailandensis* (229–232). Notwithstanding its underacylated, heterogeneous tetra‐ and penta-acylated lipid A (233), LPS isolates from *B. cepacia, B. dolosa, B. cenocepacia, B. mallei*, and *B. multivorans* were reported to potently induce TLR4 mediated NF-κB signalling (234–237). The molecular background for a robust induction of the pro-inflammatory signaling by underacylated Ara4N‐modified *Burkholderia* lipid A/LPS isolates is not yet clarified, particularly, because only hexaacylated lipid A patterns with fatty chain length 12-14 carbon atoms are known to elicit efficient TLR4‐mediated responses (183). Whether the presence of Ara4N modification at both phosphate groups renders *Burkholderia* LPS to a strong TLR4 agonist is not yet proven, although mono-substitution of P-1 with Ara4N (1–O–P–β-l-Ara4N) did not significantly enhance the cytokine-inducing capacity of synthetic *Burkholderia* lipid A *in vitro* (182).

Since penta-acyl *Burkholderia* lipid A is structurally “unsuitable” to function as potent TLR4 agonist, it is rational to assume that other LPS sensing proteins could be responsible for recognition of *Burkholderia* LPS patterns. Indeed, it has been reported that caspase-11 activation by *B. thailandensis* and *B. pseudomallei* protected mice from lethal infection outcome (135). Newest reports demonstrated caspase-11 promoted cell death induced by wild type *B. thailandensis* (lipid A is penta-acylated, modified with Ara4N), whereas tetraacylated mutants lost the ability to activate TLR4 and had 30% lower capacity in induction of caspase-11 dependent pyroptosis (229). Infection with *B. thailandensis* triggered caspase-1 mediated release of IL-1β and IL-18 and caspase-11 induced activation of the NLRP3 inflammasome leading to death of infected lung epithelial cells by pyroptosis in mice (238, 239). Further studies revealed species-specific differences in the activation modalities of caspase-11 and caspase-4 by *B. thailandensis*. In rodents, the activation of caspase-1 provoked the release of IL-18 which, in turn, induced IFN-γ to prime caspase-11 activity, whereas caspase-4 transgenic mice did not necessitate IFN-γ priming upstream of caspase-4 to control the infection (240). The significance of caspase-4 activation implicated in the formation of autophagosomes was also confirmed for *B. cenocepacia* infected macrophages (241). Thus, TLR4 seem not to belong to the primary PRR able to sense penta-acylated *Burkholderia* LPS, rather this function is taken over by the cytosolic LPS receptors such as caspase-4/11 and GBP, or other not-yet-identified proteins.

## Conclusion

The LPS induced TLR4-mediated signaling and caspase-4/11 activation drives the assembly of inflammasomes and contributes to development of inflammation, thus, mounting a beneficial defensive host immune response against infectious challenge. Juxtapose, in the conditions of unresolved inflammation, TLR4 and caspase-4/11 activation can result in the amplified innate immune signaling, systemic overexpression of the pro-inflammatory mediators and pyroptosis which prompts the onset of sepsis syndrome and a fatal septic shock (15, 22, 38, 88).

Whereas LPS-induced TLR4 complex dimerization results in the expression and release of MyD88- and TRIF-dependent cytokines such as TNF-α and interleukins activation of inflammatory caspases-4/11 by LPS arbitrates the release of IL-1β and IL-18, Gasdermin-mediated pyroptosis and is associated with high lethality (242, 243). *In vivo*, activation of caspase‐11 has been shown to provide protection against bacterial infections (135), but also to cause morbidity and mortality in a mouse model of endotoxemia (28, 34). Thus, inhibition of both TLR4 and caspase‐4/11 activation could provide instruments to control acute inflammation and to reduce the LPS-induced toxic effects. Concurrently, coordinated induction of the pro-inflammatory signaling *via* TLR4 and/or caspase-4/11 pathways is believed to mount an advantageous immune activation aimed at protection from infection and management of chronic inflammation. Thus, modulation of the innate immune responses by application of TLR4 and/or caspase-4/11 agonists or partial agonists could be a promising therapeutic approach.

Although inflammatory caspases-4/11 can directly bind the lipid A moiety of LPS, the precise molecular mechanism and the structural basis for this recognition is not yet fully understood. Caspase-4/11 exhibit somewhat different requirements to the structure of LPS compared to the TLR4 complex and seem to be more receptive to the number of phosphate groups decorating the glucosamine backbone of lipid A than to the acylation pattern, especially in respect to number and length of acyl chains. LPS-remodeling resulting in decoration of the phosphate groups of lipid A with positively charged appendages has not yet been specifically addressed in biochemical and structural studies of caspase-4/11 ligand specificities, however, our analysis suggests that these modifications could be essential for LPS/lipid A sensing by inflammatory caspases and related proteins such as GBPs. Importantly, the well-known species-specific differences in sensing lipid A variants by human and mouse TLR4 also seem to apply for caspase-4/11. For instance, caspase-4 displays much broader reactivity in sensing underacylated LPS compared to caspase-11, which might have important consequences for translation *in vivo* studies to clinical trials. Although the structural basis of lipid A/LPS recognition by inflammatory caspases is not yet completely defined, and many questions still remain unanswered, further studies will certainly decipher particular molecular signatures conferring LPS responsiveness to caspase-4/11.

## Author Contributions

AZ and HH have written the manuscript. All authors contributed to the article and approved the submitted version.

## Funding

Financial support from Austrian Science Fund FWF grants P-28915 and P-32397 is gratefully acknowledged.

## Conflict of Interest

The authors declare that the research was conducted in the absence of any commercial or financial relationships that could be construed as a potential conflict of interest.
