# Corrigendum: Lipopolysaccharide Recognition in the Crossroads of TLR4 and Caspase-4/11 Mediated Inflammatory Pathways

**DOI:** 10.3389/fimmu.2021.649442

**Published:** 2021-01-28

**Authors:** Alla Zamyatina, Holger Heine

**Affiliations:** ^1^ Institute of Organic Chemistry, Department of Chemistry, University of Natural Resources and Life Sciences, Vienna, Austria; ^2^ Research Group Innate Immunity, Research Center Borstel—Leibniz Lung Center, Airway Research Center North (ARCN), German Center for Lung Disease (DZL), Borstel, Germany

**Keywords:** lipid A, inflammation, chemical structure, innate immunity, molecular recognition, TLR4/MD-2, inflammasome, aminoarabinose

In the original article “Muzio, M., Ni, J., Feng, P., and Dixit, V.M. Science (1997) 278(5343):1612–1615” was not cited in the article.

The citation has now been inserted as reference “51” in section L**ipopolysaccharide Detection by the Innate Immune System**, subsection **TLR4-Mediated Signaling Pathways**, *paragraph two*, and should read:

*“MyD88 is a 296 aa adaptor protein containing two major domains: a C-terminal TIR domain which associates with other TIR domain-containing proteins and a N-terminal death domain (DD) which mediates the interaction with the IRAK (IL-1R associated kinase) family kinases (49–51). Whereas recruitment to TLR4 is facilitated by homotypic TIR domain interactions and requires the bridging adaptor MAL (52, 53), DDs are used to engage members of the IRAK family to the complex. The whole complex comprises of 14-16 MyD88 and IRAK1, -2, and -4 molecules and has been termed the myddosome (54).”*

All reference numbers have been updated accordingly.

The authors apologize for this error and state that this does not change the scientific conclusions of the article in any way. The original article has been updated.

